# Mental health pluralism

**DOI:** 10.1007/s11019-024-10233-8

**Published:** 2024-11-13

**Authors:** Craig French

**Affiliations:** Department of Philosophy, University of Nottingham, Nottingham, UK

**Keywords:** Mental health, Pluralism, Monism, Well-being, Philosophy of health, Health, Positive psychology

## Abstract

In addressing the question of what mental health is we might proceed as if there is a single phenomenon—mental health—denoted by a single overarching concept. The task, then, is to provide an informative analysis of this concept which applies to all and only instances of mental health, and which illuminates what it is to be mentally healthy. In contrast, mental health pluralism is the idea that there are multiple mental health phenomena denoted by multiple concepts of mental health. Analysis and illumination of mental health may still be possible, but there isn’t a single phenomenon or concept to be analysed in addressing the question of what mental health is. The question of pluralism has been overlooked in the philosophy of mental health. The discussion to follow is an attempt to get us to take mental health pluralism seriously. To that end, in this essay I have three primary goals: (1) to give a precise account of what mental health pluralism is, (2) to show that the question of pluralism should not be neglected in debate about what mental health is, and (3) to argue for mental health pluralism. I also draw out some implications of this discussion for philosophy, science, and psychotherapy.

## Introduction

In addressing the question of what mental health is, or of what it is to be mentally healthy, we might proceed as if there is a single phenomenon—mental health—denoted by a single overarching concept. The task, then, is to provide an informative analysis of this concept which applies to all and only instances of mental health, and which illuminates what it is to be mentally healthy. In contrast, *mental health pluralism* is the idea that there are multiple mental health phenomena denoted by multiple concepts of mental health. Analysis and illumination of mental health may still be possible, but there isn’t a *single* phenomenon or concept to be analysed in addressing the question of what mental health is.

In many areas of philosophy, pluralist vs anti-pluralist stances are vigorously debated, and forms of pluralism are fruitfully pursued. But the question of *mental health* pluralism has been overlooked.^1^ This is surprising. For there are several different conceptions of mental health considered in mental health studies. Vaillant (2012), for instance, distinguishes seven: ranging from mental health as maturity, to mental health as socio-emotional intelligence, through to mental health as subjective well-being.^2^ We might naturally wonder, then, whether these are competing conceptions of the same phenomenon, mental health, or instead different conceptions of different mental health phenomena.

Moreover, there is huge variation in everyday thought and talk. Compare someone who extols the virtues of regular exercise and an improved diet as good for their mental health with someone who speaks of mental health improvements after being hospitalised with heavy doses of medication to tackle psychosis. Compare Broadmoor Hospital, a high security psychiatric hospital in the UK which “treats” men detained under the 1983 Mental Health Care Act, with universities that provide puppy therapy to help students with their mental health. Finally, compare cultures steeped in biomedical thinking about health with African subcultures that view health as more social than biological, and as part of an ‘entire magico-religious fabric’ (Lambo 1964, pp. 446–447).^3^ Does ‘mental health’ mean the same thing across such radically different contexts? Maybe. But this is not obvious.

Such academic, individual-level, institutional-level, and sociocultural variation provides *prima facie* motivation for at least *considering* mental health pluralism. Whether ultimately we should *accept* mental health pluralism requires further reflection, but the neglect of the question of pluralism in the philosophy of mental health is unjustified. Thus, in what follows, I foreground the question of pluralism, and argue that we should take mental health pluralism seriously. To that end, I have three primary goals:

(I) To offer an account of what mental health pluralism is (section “What is mental health pluralism?”).
(II) To argue that the question of mental health pluralism is relevant to debate about what mental health is (section “The neglect of the question of pluralism”).
(III) To argue for mental health pluralism (section “An argument for mental health pluralism”).

I’ll connect each of these to work in the philosophy of well-being. First, the account of pluralism I develop in section “What is mental health pluralism?” is modelled on the account of well-being pluralism offered by Mitchell and Alexandrova (2021).

In section “The neglect of the question of pluralism” I consider the discussions of Keller (2020) and Wren-Lewis and Alexandrova (2021) which grapple with the question of whether we can identify mental health with well-being. I use these recent discussions to illustrate the importance of relating discussions of what mental health is to the question of pluralism. This section will, I hope, highlight the need for proponents of views about the nature of mental health to clarify their commitments by addressing whether or not they should be understood in pluralist terms.

Finally, in section “An argument for mental health pluralism” I present an argument for pluralism that has structural similarities to the argument for well-being pluralism in Alexandrova (2017). What I call the *variation argument* is simply that certain ways in which our mental health ascriptions vary across contexts are best explained by pluralism. Though I aim to support mental health pluralism in this section, more minimally I hope that it will at least prompt proponents of views about the nature of mental health who accept an anti-pluralist stance to make explicit, and defend that stance.

In the conclusion, I draw out some implications of pluralism for philosophy, science, and psychotherapy (section “Conclusion”).

Given that the question of pluralism has been neglected in existing debate about what mental health is, my strategy in what follows will not be to outline and then react to existing anti-pluralist views. The discussion will be more constructive than reactive. Anti-pluralist stances will appear either in an abstract way, or as potential interpretations of existing views, but the primary goal is the construction of a new pluralist picture, as opposed to the assessment of existing anti-pluralism.^4^

## What is mental health pluralism?

With mental health, there is much plurality: in problems, treatments, and institutions; in routes to mental health; in developmental phases of mental health. And there are various areas of science that are concerned with mental health, and numerous models and measures of mental health.^5^

Acknowledging such plurality doesn’t make one a ‘mental health pluralist’ in the sense I intend. For this plurality is consistent with there being a single substantive thing, *mental health*, which these different problems etc. are concerned with. Mental health pluralism denies that there is a single substantive phenomenon, mental health, and instead holds that there are multiple phenomena, depicted by multiple concepts of mental health.^6^

In this section I’ll make this more precise by applying the framework for understanding well-being pluralism developed by Mitchell and Alexandrova (2021).

### Conceptual pluralism about well-being

Motivated by the idea that we seem to have radically different things in mind in ascribing well-being in different contexts, what Mitchell and Alexandrova call *conceptual pluralism* about well-being entails two claims: anti-essentialism, and contextualism: ‘there is no single essence which characterises all and only instances of well-being’, but instead, ‘there are many different, inconsistent concepts of well-being, which are appropriately invoked in different contexts and at different times’ (2424). In contrast, *conceptual monism* denies both claims, endorsing instead essentialism, and anti-contextualism.

The pluralist doesn’t have to deny that there is *anything* in common to all instances of well-being. Mitchell and Alexandrova even suggest that all ascriptions of well-being may relate to how someone is doing or fairing (2426). However, the pluralist insists that such features aren’t sufficient to capture the *essence* of well-being.

Consider one of Mitchell and Alexandrova’s helpful examples: Suppose that a palliative care nurse speaks of how a certain treatment plan will improve the well-being of patients who are near to death, and an urban planner speaks of how aspects of a new plan for the town square layout will improve the well-being of members of the public. The pluralist will hold that when the nurse and the planner speak of well-being, their very different contexts determine that they mean different things. For given that palliative care and urban planning involve very different practical goals and contraints (e.g., supporting someone’s quality of life in the face of serious illness vs supporting people to access local amenities), they are practices which involve substantial differences in what is relevant to well-being and in the thresholds for well-being.^7^ As Mitchell and Alexandrova note ‘the palliative care nurse might be more likely to use a general measure of subjective well-being or life satisfaction, whereas the urban planner might be better served by an objective measure, which takes into account access to parks, public services, sports facilities, and so on’ (2425).

The pluralist need not deny that there is *a* common core to what ‘well-being’ means across these contexts (e.g., to do with how people are doing or fairing). But this common core is thin, and not capable of capturing the *essence* of well-being. For the pluralist, there is no such essence.

### Conceptual pluralism about mental health

Looked at in one way, mental health monism and pluralism are simple: the monist thinks that mental health is a single phenomenon, denoted by a single concept, whereas the pluralist denies this, and holds that there are multiple mental health phenomena denoted by multiple concepts of mental health.^8^ These positions are more complex when explained in terms of Mitchell and Alexandrova (2021)’s framework—since on this framework monism and pluralism are positions that meld conceptual, linguistic, analytical, and metaphysical committments. But before we come to this, I want to pause to consider a notion that figures in this framework: essence.

Mitchell and Alexandrova (2021) take conceptual monism about well-being to hold that there is an essence to well-being that applies to all and only cases of well-being—something denied by the pluralist. I think we should be cautious in taking this up in our formulations. For on a common understanding, the *essence* of something is that thing’s true mind-independent nature, out there to be discovered and characterised.^9^ The problem is that with this understanding of essence in place, positions that should count as forms of mental health monism cannot count. For instance, suppose that one thinks that mental health is a single phenomenon, denoted by a single concept, and that it can be analysed in terms of non-trivial context-insensitive conditions that apply to all and only cases of mental health. But suppose also that it is an entirely *mind-dependent* phenomenon (e.g., entirely interest- or purpose-relative).^10^ Well, then mental health won’t have an essence on the common understanding.

So, in applying Mitchell and Alexandrova (2021)’s framework, I think we should not follow the common understanding of essence. But this doesn’t mean that we should reject essence talk. Part of what such talk is trying to capture, in the case of well-being monism, is the idea that we can ‘identify...[something] that characterises all and only instances of well-being, and so provide a clear definition of well-being which applies exhaustively across all contexts’ (Mitchell and Alexandrova, 2416). We can preserve this in our formulation of mental health monism, and still use the terminology of essence to capture it, so long as we don’t insist that what is thus identified must be *mind-independent* conditions. Instead, I propose that we understand the claim that mental health has an essence simply as the claim that there are non-trivial context-insensitive conditions that apply to all and only cases of mental health. To give the essence of mental health—to define mental health, or say what mental health *is*—is just to articulate these. It is a further question whether such conditions are mind-independent.

With this in place, we can understand our positions more fully as follows:

*Mental Health Monism:*

There is just a single concept of mental health which ascriptions of mental health express regardless of context. There is just a single phenomenon of mental health, denoted by this concept. There is an essence of mental health given by a set of non-trivial context-insensitive necessary and sufficient conditions that exhaustively characterises this single phenomenon/concept.

*Mental Health Pluralism:*

There is not just a single concept of mental health that mental health ascriptions express, rather the content of mental health ascriptions is context-sensitive such that different concepts of mental health are expressed in different contexts. Mental health is not a single phenomenon, there are multiple mental health phenomena. There is no essence to mental health.^11^

People can take radically different paths on the journey to good mental health. The monist can admit this, it is just that they will insist that the destination, mental health, is the same in all cases.

Similarly, what constitutes mental health can be different for different people, in different circumstances, and at different times. As we’ll see in section “An argument for mental health pluralism”, the monist can admit this. It is just that they will insist that what is thus made up differently—mental health—is the same in all cases.

Distinctively, the pluralist admits a further sense in which we can be mentally healthy in different ways: two individuals can both be in states of good mental health, and yet these states be radically different in that there is no essence that unites them.

To illustrate, consider again someone who speaks of improvements to their mental health from exercise and healthy eating. Here they might emphasise mood and mental energy. Now compare this to someone who speaks of improvements to their mental health after a period of medication. Here they might emphasise reduction in incidences of psychosis.

The pluralist denies that there is an essence that captures both instances. They will emphasise that different concepts of mental health are in play across these contexts. In the first case, the focus is on good mood and liveliness (positive psychological states), in the second, ameliorating psychosis (the absence of (so-called) mental disorder). After steps toward improvement, these individuals are both mentally healthy, but *not in the same sense*. The different contexts determine different meanings for ‘mental health’.

Now, the pluralist doesn’t have to deny that there are *any* shared features, covering each instance. But they will insist that any such common core will be very thin (e.g., relating to improved mental capacities), or itself context-sensitive. This means that it will be inadequate as an analysis of the *essence* of mental health (for more on this, see section “Development”).

So far, I’ve set out more precisely the core commitments of mental health pluralism—and its rival, mental health monism. More details and refinements will emerge as the discussion develops below. In particular, we will further explore a core distinction between monism and pluralism: namely, that monists reject, whereas pluralists accept, the context-sensitivity of mental health ascriptions. That is, monists hold that there is a single stable meaning of ‘mental health’ operative across different contexts, whereas pluralists hold that contextual differences can determine different meanings of ‘mental health’. However, as we’ll explore below, a more sophisticated ‘concessive’ monist can maintain that even if contextual differences do not determine differences in the meanings of mental health ascriptions, they *can* determine differences in the kinds of things that make mental health ascriptions true. Later we will consider the extent to which this can help the monist to capture the variation and plurality that motivates pluralism in the first place (section “An argument for mental health pluralism”).

Before we turn to this, I want to illustrate the relevance of the question of pluralism by arguing that its neglect in the philosophy of mental health is problematic.

## The neglect of the question of pluralism

If one sets out to understand what mental health is, and accepts monism, then the kind of enquiry one engages in is an enquiry into a single phenomenon, mental health. Whereas if one accepts pluralism, then one is faced with trying to understand a potentially bewildering variety of phenomena. At a general level, then, the question of pluralism should not be neglected in debate about what mental health is: as the stance one takes on pluralism will shape the very project one is engaged in in trying to understand mental health.

Foregrounding the question of pluralism is particularly important if we approach the question of how to understand mental health by engaging in a prominent form of philosophical analysis, where the central concern is with how to define things in more basic terms. Projects in this mould make claims of the form *mental health can/cannot be identified with/analysed/defined in terms of* *x* (e.g., the absence of mental disorder, the presence of certain psychological goods). I’ll argue that unless we relate claims of this form to the question of pluralism, they will be inherently unclear or ambiguous. The question of pluralism should thus be addressed in philosophical debate about what mental health is.

I’ll illustrate these points by examining some recent literature which is concerned with the philosophical analysis of mental health: the discussions of Keller (2020) and Wren-Lewis and Alexandrova (2021) which consider whether mental health can be identified with well-being.

### Background

The aforementioned articles both move beyond a *negative* definition of mental health (as the absence of mental illness, disease, disorder and the like). Such a negative definition is implicit in a lot of psychiatric discourse (see Tengland 2001, p. 15) and a version of it is defended by Boorse (1976). But, as Wren-Lewis and Alexandrova argue, it is too thin or undemanding as a definition of mental health since ‘there is more to mental health than the absence of mental illness’ (690). Likewise, Keller (2020)’s focus is explicitly on ‘positive mental health’, understood as ‘the presence of certain mental goods: certain mental skills, habits, and capacities’ (228).

But what might mental health be if it is not merely the absence of mental disorder? How should we understand the ‘mental goods’ that are definitive of positive mental health? Here we will focus on some positive definitions of mental health that we find in public discourse and academic literature that answer this question by identifying mental health with *well-being*. The articles I am focusing on consider two important sources for this: (1) the World Health Organisation’s (WHO’s) definitions of health and mental health, and (2) definitions in positive psychology.

The WHO define mental health as ‘a state of mental well-being that enables people to cope with the stresses of life, realize their abilities, learn well and work well, and contribute to their community’.^12^ As Wren-Lewis and Alexandrova point out, this fits well with the WHO’s positive understanding of health more generally, as a ‘state of complete physical, mental and social well-being and not merely the absence of disease or infirmity’.^13^

Regarding positive psychology, Keller (2020, p. 229) highlights an influential definition of mental health given by Huppert and So (2013). Here the authors equate mental health with well-being, and as Keller notes, they take well-being to consist in ‘competence, emotional stability, engagement, meaning, optimism, positive emotion, positive relationships, resilience, self-esteem, and vitality’. Citing Keyes (2013), Keller adds that in positive psychology the ‘identification of positive mental health with well-being is so pervasive that it is often taken for granted’ with authors using terms such as ‘mental health,’, ‘well-being,’ and ‘mental well-being’ interchangeably.

Both Keller and Wren-Lewis and Alexandrova reject such attempts to define mental health in terms of well-being. What, then, are their arguments?

### The arguments

I’m going to look at four arguments against identifying mental health with well-being. Though different, they have shared components. Namely:

- An articulation of a certain way of thinking about mental health or a person’s mental health (the ‘MH component’),
- an articulation of a certain way of thinking about well-being or a person’s well-being (the ‘WB component’), and there are
- considerations which support the disassociation of (the person’s) mental health and well-being in the relevant senses (the ‘Disassociation Component’).

Thus:

1. *The Positive Mental Health without Well-Being Argument:* Keller considers someone whose well-being is poor because they are a victim of a natural disaster (*WB Component*). The victim of the natural diaster may have good mental health in that they cope with the ‘misfortune in the most mature and constructive manner possible. Her emotions and feelings and ways of dealing with her situation may be as healthy as can be’ (230) (*MH Component*). Thus poor well-being doesn’t entail poor mental health (it is perfectly consistent with strong positive mental health), and so there is a mismatch in well-being and mental health (*Disassociation Component*).
2. *The Demandingness Argument:* Wren-Lewis and Alexandrova (691–692) argue that positive definitions of mental health can be too demanding. They suggest that this charge applies to the WHO definition of mental health. The argument first considers the understanding of well-being that the WHO uses to define mental health (*WB Component*), and then draws out in several ways how this results in a demanding definition of mental health. First, it sets a high bar for being mentally healthy (e.g., in terms of realising one’s potential), second, it demands that mentally healthy individuals actually meet such standards, not just *think* they do, and third it demands conformity to specific modern norms, such as not being a burden to one’s community *(MH Component)*. But since we think that people can be mentally healthy without satisfying such ideals, we have a mismatch between mental health and well-being along the dimension of demandingness *(Disassociation Component)*.^14^
3. *The Well-Being without Positive Mental Health Argument:* In another argument, Keller asks us to consider someone whose issues with impulse control lead them to (impulsively) eat foods that are good for them, someone whose social phobia leads them to structure their social interactions in a way that are really enjoyable, or someone whose alcoholism leads them to friendships and support networks that are very good for their well-being (231). In each case we can understand this person as having some mental health problem which means that they have poor mental health (*MH Component*). Yet they also have high well-being as afforded by mental goods like enjoyment, and social connectedness *(WB Component)*. In such cases, mental health *deficits* lead to well-being *gains*, meaning that again there is a mismatch between mental health and well-being *(Disassociation Component)*.
4. *The Medicalisation Argument:* Focusing on positive psychology, Wren-Lewis and Alexandrova note that setting ‘a given level of a positive good [e.g., happiness] as a condition of mental health turns this good into a medical category when it was not previously so’ (692–693). The flip side of this is that the corresponding negative state (e.g., unhappiness) then gets uneasily transformed into a medical problem. The authors note that such medicalisation is socially and politically suspect (693). So, this argument focuses on conceptions of well-being in terms of specific positive emotions and highlights how these aren’t usually understood as medical in character (*WB Component*). However, on an ordinary understanding, mental health is medical in character (*MH Component*). There is therefore a mismatch between mental health and well-being along the dimension of medical character (*Disassociation Component*).

### Discussion

At one level our arguments are clear: for they all aim to show that we should not identify mental health with well-being. However, once we foreground the question of pluralism—a question that is not on the table in these discussions—matters aren’t so clear.^15^ For we can then ask questions such as: Do our arguments exclude pluralism? If not, are they claiming that, of the potentially many forms of mental health, *none* of them can be identified with well-being, or just that *not all* of them can be? Asking such questions of our arguments yields significantly different interpretative options, particularly:

(A) There is just a single form of mental health, and it cannot be identified with well-being.
(B) There may be multiple forms of mental health, but none of them can be identified with well-being.
(C) There may be multiple forms of mental health, but not all of them can be identified with well-being.

According to (A), our arguments commit to an anti-pluralist, monist framework. The arguments aim to show that the single substantive phenomenon, mental health, cannot be identified with well-being. Whereas with (B), our arguments don’t commit to monism, but nor do they commit to pluralism. They are *pluralist friendly* in that they are open to the possibility of there being multiple forms of mental health. The arguments aim to show that even if there are multiple forms of mental health, none of them can be identified with well-being. (A) and (B) are thus united in that they both attempt to establish that *no* form of mental health can be identified with well-being—a particularly strong form of the ‘mental health cannot be identified with well-being’ claim.

Interpretation (C) is similar to (B) in being pluralist friendly, yet it is different to (A) and (B) in that its non-identity conclusion is weaker: it commits only to the claim that not all forms of mental health can be identified with well-being. This leaves it open that some forms of mental health *can* be identified with well-being.

In terms of how they are formulated, our arguments are ambiguous when it comes to these interpretive options. It is only by foregrounding the question of pluralism that we can fully understand these arguments, for only then can we appreciate the above interpretative options, and work towards resolving the question of how precisely the arguments are to be interpreted.

To consider this last question, we need to move beyond issues of formulation, and look at the substantive considerations that these arguments involve. Now suppose we do this, and it turns out that these arguments are best understood in terms of (A), which rejects pluralism. Even then, we won’t have undermined the main claim of this section: since the claim is not that we should *accept* pluralism, but that the question of pluralism should not be neglected in debate about what mental health is. And explaining how our arguments involve substantive considerations best understood in terms of (A) *supports* this. For, just like the pluralist friendly interpretations (B) and (C), the monist interpretation (A) only makes sense as an interpretation of our arguments in the context of the questions that arise once we consider pluralism.

In any case, I now want to suggest that these arguments are best understood in terms of a *pluralist* friendly interpretation anyway, namely (C). This bolsters the claim that we can only fully understand these arguments by relating them to the question of pluralism. (It is also, I hope, an independently interesting observation. As it means that our arguments are more limited in scope than they might be taken to be. To put it another way, if these arguments are intended to support stronger conclusions, they fall short).

For (A) to fit our arguments, they would have to contain considerations to support monism. And for (B) to fit, they would have to contain considerations to support the idea that the ascriptions of mental health or claims they make about mental health apply with respect to any legitimate conception of mental health (supposing there are many), not just those contained within their MH components (call this claim, *generalisability*). Yet our arguments aren’t powerful enough to support either monism or generalisability. Each argument is powerful enough to show only that *in the sense explicitly articulated in its relevant MH component*, mental health cannot be identified with well-being. This is consistent with pluralism, and the denial of generalisability—and even with the idea that some forms of mental health can be identified with well-being.

We can bring these points out more concretely by considering what a pluralist who rejects generalisability might say about each of our arguments, and how the substantive considerations inherent within the arguments don’t exclude such treatments.

Consider, then, the victim of a natural disaster. Let’s agree that their ability to cope with misfortune in a maximally mature and constructive manner, the fact that their ways of dealing with the situation are healthy, and the fact that their emotions and feelings are healthy, all indicate that the person has good mental health. We can also agree that such mental health cannot be understood simply in terms of well-being: the person in this situation has poor well-being.

However, there may be *another* sense in which their mental health is poor, and which does line up with poor well-being. So, consider the mental suffering, sense of loss and misery, of uncertainty and anxiety, that will be involved in the person’s mental well-being being poor in the scenario. It makes just as much sense to think of such things in terms of mental health, as it does to think of the things that Keller notes in terms of mental health.

This draws on the more general point that when we consider health, there can be significant variation in what we are interested in. In one obvious sense a person with a broken ankle is in a state of poor physical health. In another sense, they might at the same time be in excellent physical health: for they are healing perfectly well, with no complications or structural issues impeding recovery, and no other physical health problems. If our focus is on trauma and symptoms, we see poor physical health, if our focus is on the broader physical capacities, reaction, coping, and development, we see good physical health.

Similarly, sometimes when we are interested in mental health, our focus is on something akin to trauma or wound, and the symptoms of that. This can include the experience of the individual, how painful it is, how much suffering there is. But at other times it is on broader capacities and abilities to cope, and the appropriateness of responses to trauma. The individual in Keller’s scenario is mentally wounded, and experiencing great suffering. In this respect they are in poor mental health—and may understandably seek support from mental health services in getting through this. Yet in another sense they are mentally healthy, for the misery they experience is appropriate to the circumstances, and the trauma they have suffered has not disrupted their underlying capacities to think, feel, function, and cope.^16^

Thus, we can grant that the Positive Mental Health without Well-Being Argument supports the idea that the person has good mental health. And that in this sense, good mental health is not aligned with well-being. Still, this argument doesn’t exclude the idea that there is another legitimate sense of ‘mental health’ that applies in this scenario too, on which the person has poor mental health (and where this *is* aligned with poor well-being).

We can draw a similar conclusion about the Demandingness Argument. We can agree that people can be in good mental health without having well-being according to the WHO’s demanding conception of mental health/well-being (e.g., the person described in Keller’s scenario). But for all Wren-Lewis and Alexandrova’s considerations show, perhaps we do sometimes *also* understand mental health in more WHO-like ideal terms.

For instance, if our context is one of reflecting upon population-level trends in mental health, it may make sense to operate with a WHO-like ideal notion. Consider a national health service trying to measure the effectiveness of certain mental health services. Take for instance adult men aged 30–39 who were made redundant owing to the recent COVID-19 pandemic, and suffered mental health problems associated with all of this, but who accessed mental health services for support, including psychological support around finding work again in light of what they have suffered. It may make sense to measure their progress in terms of the demanding WHO standards of coping, community engagement, learning and work, and conceive of this in terms of mental health.

This would be problematic—and *overly* demanding—if we were trying to capture all of the ways we think about mental health. For it would not sit comfortably with the other areas of our thought and talk where we don’t understand mental health in such demanding terms (e.g., certain therapeutic contexts). But it is not problematic if we regard this conception of mental health as merely one amongst others.

Now take the person with a social phobia. We can agree with Keller that this can be understood as a lack of mental health: indeed, such a person may even meet criteria for diagnosis of a mental disorder. Yet since there are positive aspects of this person’s mental well-being, which leads them to flourish, and be happy, we might hold that they also, in another sense, have mental health.

Again this reflects more general ways of thinking about health wherein we recognise that there can be *health within illness*. There is helpful discussion of this in Carel (2019, pp. 96–97) who describes studies of people with chronic illness and/or disabling conditions who nonetheless rate their health as excellent.^17^ This makes sense if, as Carel urges, we recognise the ‘multidimensionality of the concept of health’ (96), and within this take seriously dimensions which invoke not simply what is going on biologically in the body but also subjective experience, including experiences of healthiness and well-being (97). For then we can recognise the co-existence of illness with forms of health that are to do with one’s experience of living, which may involve experiences of enjoyment, contentment, opportunity, and the power of the self. A pluralist perspective on this holds that someone can lack health in one sense (e.g., have chronic illness), yet at the same time have excellent health, in another, more subjective, sense.

So, for all the Well-Being without Positive Mental Health Argument says, this is a scenario where in one sense one lacks health (taking social phobia to be a disorder), yet in another sense one has health—and this can be partly understood phenomenologically in terms of one’s experience of well-being as it occurs in the way one enjoys the kind of social life one has constructed in light of one’s social phobia, and the way one experiences the power of one’s self manifest within this.^18^

Finally, consider the Medicalisation Argument. We can agree that some of our concepts of mental health are medical in character. But perhaps *others* aren’t. Keller notes that ‘People commonly talk about making lifestyle changes, ending relationships, taking days off work, and leaving Facebook for the sake of their mental health, without having thoughts of avoiding mental disorder’ (228). Here Keller’s point is to dissociate concern with mental health with concern with psychopathological notions like mental disorder. However, this also highlights how we don’t always think about mental health in *medical* terms. For presumably the people Keller describes here are also not having thoughts of any medical improvements. It is, admittedly, hard to specify precisely what we mean by the medical. But presumably we can imagine people of the sort that Keller describes who think that leaving Facebook helps their mental health but also that it is *not* a medical matter.

So, though there is a concept of mental health (or various concepts thereof) that relate to medicine, for all the medicalisation argument says, there are also non-medical concepts of mental health that we appeal to in our variegated thought and talk. There is no problem of medicalisation, then, in accounting for any of *those* concepts in terms of specific positive emotions such as happiness.

Our arguments contains claims about mental health *as understood in their relevant MH components*. By taking the perspective of a pluralist who rejects generalisability, we can see that none of them contains considerations to support the idea that their MH component articulates the single substantive concept of mental health. Nor do they contain considerations to support the idea that their ascriptions of mental health or claims about mental health apply beyond the conceptions of mental health contained within their MH components.

And even though our arguments support the claim that *certain* forms of mental health cannot be understood in terms of well-being, for all they establish, *other* forms of mental health can.

My point is not that we must accept the perspective of the pluralist who denies generalisability, and the particular treatments of the arguments I’ve outlined from that perspective. The point is that the arguments *as they stand* don’t exclude that perspective or those treatments.

To return to the main theme of this section, I’ve argued, by considering some concrete examples, that we should not neglect the question of pluralism in philosophical debate about what mental health is. I’ve argued that we cannot properly understand the central arguments of some recent work unless we relate them to the question of pluralism. For only once we foreground this question can we appreciate the interpretive possibilities. Though I’ve illustrated this with respect to the claim that mental health cannot be identified with well-being, the point applies more generally. Any claim of the form ‘mental health can/cannot be identified with *x*’ will be inherently unclear or ambiguous unless it is explicitly related to the question of pluralism. Since for any such claim, we can ask whether it is to be understood in anti-pluralist terms or pluralist friendly terms—and if pluralist friendly, whether it applies to all or just some forms of mental health (assuming there are multiple forms).^19^

## An argument for mental health pluralism

So far, I’ve offered an account of mental health pluralism, and argued that we should take the question of pluralism seriously in debate about what mental health is. In this section, I’ll argue for pluralism. What I’ll call the *variation argument* is simply that certain ways in which our mental health ascriptions vary across contexts are best explained by mental health pluralism.^20^

### A vignette

Fabio has long been keen on having a healthy mind and body. A regular gym-goer and sport lover, with a personal trainer-*cum*-life coach. Unfortunately, Fabio suffered a physical injury that meant he was unable to train or play football for several months. This had a significant impact on his life: he felt glum, frustrated, limited, and lacklustre. He decided to see his trainer/coach for physical and psychological support as part of his recovery. On the psychological side, the trainer helped Fabio to bolster his mood and motivation, and to think positively about food, exercise, and sport. He also helped Fabio with perspective taking, and in celebrating his psychological strengths. This helped Fabio to feel much better.

Several years later, Fabio admitted himself into a psychiatric unit, complaining that he had been having intermittent psychotic episodes for months, but that they had increased in frequency recently. The episodes included hearing voices, experiencing persecutory delusions, and paranoia. His behaviour was increasingly out of character—e.g., he’d started having violent outbursts which he’d never had before. Eventually, he was treated for schizophrenia. This included both drug medication, and psychotherapy. Fabio suffered tremors as a side effect of his medication, and in hospital he felt a little glum, frustrated, limited, and lacklustre. However, he experienced the treatment as hugely beneficial, as it helped to stabilise him, and eliminate the disabling psychotic episodes.^21^

With this vignette set out, consider the following contexts:

- *Psychiatric Assessment*
  This is a context focused on Fabio at the end of the vignette in which Fabio and his lead psychiatrist are considering how he is getting on, and the progress he has made. The central interest in these assessments, and goal of their relationship, is Fabio’s recovery from mental disorder. Reflecting on how well Fabio is responding to the anti-psychotic medication psychologically, the psychiatrist remarks that he is in good mental health.
- *Friendly Visit*
  This is a context focused on Fabio at the end of the vignette, in which Fabio’s trainer-*cum*-life-coach visits to offer some moral support. Since working together years ago, the central concern in their relationship has been Fabio’s positive psychological well-being. Reflecting on how different Fabio is to the last time they interacted (when Fabio had recovered from his psychological dip after his injury), the trainer notes how Fabio is not in good mental health, but is confident that he can help him again.

### The variation argument

Consider then:

(M) Fabio is in good mental health.

Intuitively, (M) is true in Psychiatric Assessment, but not in Friendly Visit. And there seems to be a difference in what is *relevant* to mental health across these contexts: a difference in what factors make it the case that mental health is present or absent across these contexts.^22^ For what seems to be relevant to mental health in Friendly Visit are factors aligned with mental well-being, on some ways of understanding well-being, such as positive mood and mental energy. But such factors don’t seem to be relevant in Psychiatric Assessment, since there the central focus is on Fabio’s mental disorder status.^23^

So, reflection on our vignette supports:

(i) (M) is true in Psychiatric Assessment, but not in Friendly Visit.
(ii) There is variation in what is relevant to mental health across the contexts.

With these claims on the table, we can begin to appreciate what our options are when it comes to making sense of our vignette.

A monist might simply reject (i) and (ii) by developing an account of mental health that is so narrow that it cannot validate (i) or (ii). Since monism developed in this way refuses to assign any significance to the variation or plurality that our vignette is supposed to highlight, we can call this *obstinate monism.*^24^

The popular idea that mental health is simply the absence of mental disorder can be developed as a form of obstinate monism. Accordingly, (M) has the same semantic content in both of our contexts: that Fabio is free from mental disorder. On this view, what is relevant to mental health is simply whether Fabio has, or meets diagnostic criteria for, a mental disorder, and this doesn’t shift across our contexts. Moreover, (M) is true in Psychiatric Assessment, but *also true* in Friendly Visit—as Fabio is the same across these contexts. So, (i) and (ii) are false.

The problem with any form of obstinate monism is that (i) and (ii) are very plausible. (i) records our intuitive judgements about the truth value of (M) in these different contexts. Moreover, rejecting this claim implies that there is *widespread* error or sloppiness in our thought and talk about mental health.^25^ Understanding mental health in the positive way relevant to Friendly Visit is common in ordinary and technical discourse (as we saw earlier, a similar way of thinking is to be found in positive psychology). And understanding mental health in the negative way relevant to Psychiatric Assessment is also common in ordinary and technical discourse (consider how common it is to think of mental health in psychopathological terms in psychiatric contexts).

Now the fact that obstinate monism has such counterintuitive consequences doesn’t mean that it is false. But it does face a significant challenge: that of explaining why we should, and how we can, accept such consequences. In the absence of such explanations, and given that there are other options—even for the monist, as we’ll shortly see—we can take obstinate monism to be an option of last resort. Let’s focus, then, only on approaches that accept (i) and (ii). Two options are salient: *mental health pluralism*, and what we can call *concessive monism*.^26^

The pluralist proposes a specific way of accommodating (ii): the contexts of utterance differ in what is relevant to mental health, and what ‘mental health’ means is sensitive to such contextual differences.^27^

The context of Psychiatric Assessment is one in which what is most relevant to mental health is the absence of mental disorder, and thus the content of (M) is that Fabio’s mental health, negatively conceived, is good. And this is true. Yet in the context of Friendly Visit, what is most relevant to mental health is the presence of psychological goods such as happiness and liveliness, and disorder is not in focus. And thus the content of (M) is that Fabio’s mental health, positively conceived, is good. But this is not true. Thus the pluralist can validate (i).

What I am calling a *concessive* monist is someone who has a more concessive attitude to variation and plurality than the obstinate monist. They can accommodate (i), so long as they characterise the essence of mental health in relatively broad terms. We’ll come back to this.

But what of (ii)? Here, as discussed towards the end of section (“What is mental health pluralism?”), the monist can hold that even though mental health is a single phenomenon, it (or the lack of it) can be *constituted* or *realised* differently in different cases. So even though in both contexts a single phenomenon, mental health, is in focus, and (M) doesn’t change its meaning across these contexts, there is variation in what makes it the case that mental health is present or absent across these contexts. Such a monist holds that we can only evaluate a mental health ascription for truth relative to a context. But not because the *contents* of such ascriptions are context-sensitive, but because the *kinds of factors that can ground truth* is.^28^

So, what grounds the truth of (M) in Psychiatric Assessment is a certain state of Fabio: an absence of psychosis. Now, this very state is also present in the context of Friendly Visit. But (M) is *not true* in that context. This is because states such as this aren’t relevant to grounding the truth of mental health ascriptions in *that* context. So even though a state that is constitutive of mental health in the Psychiatric Assessment context is present in the Friendly Visit context, it is not thereby constitutive of mental health in the Friendly Visit context, because there is a shift in what factors are constitutive of mental health across these contexts.

This explains the *shape* of concessive monism. But how might it be implemented? Though not presented with these concerns in mind, the account of mental health offered by Wren-Lewis and Alexandrova (2021) will work here. Wren-Lewis and Alexandrova claim that mental health is a psychological primary good, defined in terms of capacities we have to feel, think, and act in ways that enable us to value and engage in life (696). They suggest that valuing life ‘consists in capacities to care about certain states of affairs–features of ourselves, others, and our environment’ (696). When it comes to engaging in life they understand this partly in terms of capacities that make up psychological flexibility or adaptiveness (697).

The monist could therefore propose the following as a characterisation of the essence of mental health: necessarily, *S* is mentally healthy if and only if they have good capacities to feel, think, and act in ways that enable them to value and engage in life. Call these conditions *CAP*. *CAP* are thus offered as non-trivial context-insensitive conditions that apply to all and only cases of mental health. Mental health ascriptions are context-invariant and express just a single concept of mental health, the content of which is exhausted by *CAP*. Thus, (M) is true if and only if Fabio satisfies *CAP*.

In Psychiatric Assessment the factors relevant to grounding the truth of *CAP* are to do with Fabio’s mental disorder status. When suffering psychosis, Fabio’s capacities to feel, think, and act in ways that enable him to value life in this sense are compromised, as is his ability to be psychologically flexible. When in the grip of paranoid delusions, or engaged in violent outbursts, he’s unable to care for himself, others or his environment in ways that he normally would. And he’s unable to perceive his environment in the realistic ways required for flexibility and adaption. Those capacities are restored when he is treated. We can thus understand how in Psychiatric Assessment, where it is just such factors that are relevant to the truth of *CAP*, Fabio satisfies *CAP*, and (M) is true.

Yet in the context of Friendly Visit the only factors relevant to grounding the truth of *CAP* are to do with positive psychological states. Just like when he initially suffered his sporting injury, in the hospital Fabio is experiencing a psychological dip—hazy and numb, not as happy as he would like to be, and lacking in energy. Relative to such concerns, his capacities to feel, think, and act in ways that enable him to value and engage in life are severely limited. Thus in the context of Friendly Visit, where it is just such factors that are relevant to the truth of *CAP*, Fabio fails to satisfy *CAP*, and (M) is not true.

It seems, then, that both the pluralist and the concessive monist can make sense of our vignette and our observations about it. However, I now want to argue that the pluralist has a distinct advantage here. For we can raise a challenge for the concessive monist based on the challenge that Alexandrova (2017) puts to a similar version of monism about well-being. Alexandrova argues that such a view has the very counterintuitive consequence that well-being can ‘come and go with changes in context’ or that someone can actually *improve* their ‘well-being just by changing the context of evaluation’ (20).

When it comes to concessive monism about mental health—in general—we can put the challenge like this: the concessive monist seems to be committed to a very counterintuitive consequence when it comes to characterising what happens if Fabio actually shifts between the Psychiatric Assessment and Friendly Visit contexts. Let’s spell out such a shift as follows:

**Fabio’s Shift**

Suppose that Fabio is talking to his trainer in the context of Friendly Visit. He is reminded of how similar his current feelings are to when he was in the psychological dip that his trainer helped him out of. He agrees with his trainer that he is in poor mental health. But now suppose that after the trainer leaves, the psychiatrist comes in and the context shifts so as the focus in on how much progress Fabio has made with respect to psychosis. He agrees with his psychiatrist that he is in good mental health.

Focusing now on the different *self*-ascriptions of mental health in Fabio’s Shift, it is difficult to see how the concessive monist is not committed to the claim that Fabio goes from being in a state of poor mental health, to being in a state of good mental health. For, as discussed above, in Friendly Visit (M) is not true, whereas in Psychiatric Assessment, it is true. And so Fabio’s thought, in the Friendly Visit context, that he is in poor mental health, is true, and his thought, in the Psychiatric Assessment context that he has shifted to, that he is in good mental health, is true. In this sense, it seems that the concessive monist is committed to the idea that mental health can ‘come and go with changes in context’. It seems that all that Fabio has to do to improve his mental health radically in a flash, is simply shift to a different conversational context. How can significantly changing one’s mental health possibly be so straightforward?

In contrast, the pluralist doesn’t claim that Fabio goes from being in poor mental health to being in good mental health. It is rather that he thinks about mental health in one way, and then another. In one sense, he is in poor mental health, but in another sense he is in good mental health. There isn’t a single content that Fabio entertains that is not true, and then true once he shifts contexts. Rather, Fabio is operating with different concepts of mental health at different times. He might naturally reflect upon this as well, and recognise that in one sense his mental health is poor (and he needs some of his trainer’s life coaching), yet at the same time, in another sense, his mental health is good (and he is grateful to the psychiatrist).

The difference here is that the pluralist says there is no change in Fabio’s mental health when he shifts context, whereas the monist says there is a change in Fabio’s mental health. This is highly counterintuitive, but not wanting to underestimate the ingenuity of the concessive monist, let’s frame this merely as a challenge: the concessive monist needs to either explain why, contrary to appearances, their view doesn’t have this counterintuitive consequence, or else explain why it is a price worth paying.

### Development

I’ve argued that the contextual variation outlined above is best explained by pluralism, as opposed to the versions of monism we have considered. This is a *provisional* argument for pluralism rather than a conclusive argument. For the argument has some limitations. First, it involves a restricted range of options (mental health pluralism, and two forms of monism). Given the context of this essay, these are salient options, but there may be other options we’ve yet to consider. Another limitation is that we have only considered certain dimensions of disagreement. A stronger argument will weigh pluralism against other positions along further dimensions. I hope at least that the variation argument will serve as a useful invitation to the anti-pluralist to articulate other positions, and other points of disagreement, so as the dialogue can continue.

Setting these issues aside, I want to end this section by developing the argument, and pluralism, a little further.

Since a lot of my attention has been on contextual variation, one might worry that the above discussion supports merely the linguistic and conceptual components of pluralism, and not the more metaphysical and analytical components: the idea that there are multiple mental health phenomena, and the idea that there is no essence to mental health.^29^ However, I take these components to be so tightly related that the considerations advanced support pluralism *in toto*.

The contextualism supported here helps us to appreciate how there are multiple concepts of mental health that are *expressed* in our mental health discourse. However, it has been just as much a part of our discussion to note how these different concepts actually *apply*: a positive concept of mental health truly applies to Fabio once his trainer helps him with life coaching after his sports injury; a negative concept truly applies to him after treatment for psychosis. These examples are representative of everyday and technical thought and talk. And so not only do we express different concepts of mental health in our mental health discourse, but there are different forms of mental health.

But even if there are different mental health phenomena, could they still be unified by an overarching essence? Given how we are understanding essence here, this means we will need to find non-trivial context-insensitive conditions that apply to all and only instances of mental health. Given what we’ve observed above, we can now highlight how difficult this is going to be.

In Psychiatric Assessment, (M) is true, but in Friendly Visit it is not true. Now if there are conditions which are necessary *and sufficient* for mental health, then these are conditions which must be instantiated in Psychiatric Assessment, but *not* in Friendly Visit. But it is difficult to see what such conditions could be.

A potential example here can be drawn from Wren-Lewis and Alexandrova (2021)’s suggestion that to be mentally healthy is to have good capacities to feel, think, and act in ways that enable them to value and engage in life. These conditions—*CAP*—are arguably *necessary* for mental health. At the very least they cover the two forms of mental health that we have been focusing on—negative and positive. But the pluralist can deny that they are necessary *and sufficient*. For Fabio satisfies *CAP* in Psychiatric Assessment—so (M) is true. But since Fabio is exactly the same in Friendly Visit, he must satisfy *CAP* in that context too. So, (M) must be true in that context too, contra what we have observed about Friendly Visit.^30^

What, however, if we claim that *CAP* are themselves semantically context-sensitive? Perhaps in Psychiatric Assessment, an ascription of the relevant capacities means *capacities that would be limited by mental disorder*, whereas in Friendly Visit the very same ascription means something different: *capacities that would be limited by lacking positive psychological states*. And so we no longer have a problem with the idea that *CAP* is sufficient for mental health: in Psychiatric Assessment, Fabio satisfies *CAP* *given what it means in that context*, and is therefore mentally healthy. And in Friendly Visit, Fabio is not in good mental health, but he doesn’t satisfy *CAP* in that context *given that it has a different meaning in that context*.

However, understood as such, *CAP* won’t be suitable to serve as the *essence* of mental health—given that we are understanding this in terms of context-*insensitive* conditions. Indeed, rather than aiding a challenge to pluralism, the claim that we can uphold the idea that *CAP* are necessary and sufficient for mental health by appealing to the context-sensitivity of *CAP* actually points to a way in which we might further develop pluralism. Let me explain.

Once we claim that there are multiple mental health phenomena, yet no essence of mental health, we might naturally raise the following questions: What makes these diverse phenomena all phenomena of mental health, if not the essence of mental health? How can we resist an absurd proliferation of concepts and forms of mental health, corresponding to any old ways in which we may choose to use the expression ‘mental health’? How can we uphold the intuitive idea that some—even widespread—ways of thinking and talking about mental health are just wrong?

In response to these questions of unity and constraint, the pluralist *might* insist that there is *nothing* that makes these diverse phenomena all phenomena of mental health—or at least nothing beyond our practice of grouping on the basis of perceived similarities. And similarly, they might insist that there isn’t anything metaphysically substantive to constrain conceptions of mental health—or at least nothing beyond the contingent, sociocultural choices we make in describing the world. However, I now want to suggest, further reflection on *CAP* highlights a more concessive position that the pluralist can develop to answer these questions.

For the pluralist might now say that different instances of mental health count as such only insofar as they are all instances in which *CAP*, understood explicitly as *context-sensitive* conditions, obtains: that is, different instances of mental health count as such only insofar as they are instances in which someone has good capacities to feel, think, and act in ways that enable them to value and engage in life *in some sense and to some extent*. The pluralist can even take these conditions to be necessary and sufficient for mental health, so long as they are understood as context-sensitive. This means that the pluralist can not only admit that there is unity amongst mental health phenomena, but constraint too: for any conception of mental health on which it disassociates from these conditions, will therefore not genuinely count as a conception of mental health.

Thus, the pluralist can hold that, *whatever the context*, ascriptions of mental health necessarily ascribe capacities to feel, think, and act in ways that enable one to value and engage in life. But such ascriptions also have inescapably context-determined meaning too. This mirrors the point that Alexandrova (2017, p. 22) makes about how a well-being pluralist can still acknowledge a ‘structural core’ that ‘gets filled out differently depending on circumstances’. In saying this, the pluralist denies that the *full* content of mental health ascriptions—and the concepts they express—is exhausted by *CAP* alone. It also depends on how *CAP* is determined in context (e.g., *capacities that would be limited by mental disorder*, or *capacities that would be limited by lacking positive psychological states*, or something else).

With this, the pluralist can thus preserve their core commitments: that mental health ascriptions are context-sensitive, and that there are multiple mental health phenomena denoted by multiple concepts of mental health. And although I am suggesting that such a pluralist can endorse the idea that *CAP* (understood in a context-sensitive way) are necessary *and sufficient* for mental health, as noted, this falls short of agreeing that there is an *essence* to mental health, for this requires that mental health is substantially the same across contexts.

In summary, the variation argument doesn’t *merely* help us to appreciate why we should accept the contextualism of pluralism, but also the claims that there are multiple mental health phenomena, and no essence of mental health.

Furthermore, even though the pluralist denies that there is an essence to mental health, they don’t thereby have to deny that there is *any* unity to the disparate mental health phenomena, or constraint on what counts as mental health: in particular, we’ve seen how they can embrace the idea that all and only instances of mental health are instances in which someone has good capacities to feel, think, and act in ways that enable them to value and engage in life *in some sense and to some extent*.

I am sympathetic to coupling Wren-Lewis and Alexandrova (2021)’s conditions with pluralism in this way, but *establishing* this development obviously requires further discussion. That said, even if one denies that these particular conditions are adequate to capture the common structural core of different mental health phenomena, I hope that the discussion at least illustrates how a pluralist can in theory embrace *some such* core, and therefore in principle address issues of unity and constraint without appeal to essence.

## Conclusion

I’d like to end by highlighting some theoretical and practical implications of the pluralist view I’ve been advocating.

On the theoretical side, if we accept pluralism then we may have to re-think what we are doing in giving a philosophical account of mental health.^31^ For we are not looking to analyse a single phenomenon or concept. How radical this is will depend upon the way one develops pluralism.

For all I’ve said, there may be a limited range of mental health phenomena e.g., mental health defined positively, mental health defined negatively, and a little more. Or there may be a *huge* variety of phenomena, some of which are very specific. Consider again the person who suffers from a social phobia, and who tailors their environments and social interactions because of this, but where this leads them to thrive and be happy. Explaining their situation to a friend who expresses concern about their struggle, they may feel pride in how they have adapted and describe themselves as in good mental health. But what do they mean by this? On the one hand they might mean that they satisfy a relatively broad positively defined concept of mental health. On the other hand they might (also) express more localised content: content which takes mental health to be *this* kind of adaptation in response to *these* sorts of circumstances.^32^

If we are limited-variety pluralists, then we may be able to pursue small-scale monist-like projects for each mental health phenomenon. If, however, we are wide-variety pluralists (admitting even highly specific mental health phenomena), the project of accounting for mental health may be more to do with illuminating a range of family groupings with fuzzy boundaries.

It is not just the *philosophy* of mental health that is impacted by pluralism, but the *science* of mental health too. Consider again positive psychology. Within this field there are multiple competing models and measures of mental health. A useful survey is given in Delle Fave and Negri (2021), emphasising how different positive psychological models and measures of mental health are based on different constructs of well-being. This includes psychological well-being (Ryff 1989), social well-being (Keyes 1998, 2002), PERMA—Positive Emotions, Engagement, Relationships, Meaning, and Accomplishment (Seligman 2011), and positive well-being (Huppert and So 2013). Some of these models come with their own measures of well-being or mental health (Keyes 2002, 2005).

Now, these models and measures can be interpreted differently depending on whether one accepts monism or pluralism. If one accepts monism, they can be understood as models and measures of mental health, conceived of as a single phenomenon. Whereas if one accepts pluralism they are better understood as models and measures of one *kind* of mental health phenomenon among others. Unfortunately, it is often not clear within the field of positive psychology how we should interpret the proposals. Sometimes they are offered in a way that is consistent with pluralism, whereas at other times they are offered in *favour* of alternatives that validate the idea that mental health is the absence of mental disorder (e.g., Keyes 2009).

Moreover, Delle Fave and Negri (2021) observe that there is no general consensus yet on the multiple competing models and measures to be found within positive psychology (135). They indicate that this is something to work towards in the future, and ask ‘What are the crucial theoretical, empirical, and methodological factors preventing researchers’ agreement around a unified definition of positive mental health?’ (135). However, if we take pluralism seriously, we might instead welcome this disunity as a more accurate reflection of how things are in the world of mental health. For we should be open to the idea that there isn’t just one mental health phenomenon that even positive psychologists are arguing over. And it may be that some of the aforementioned models aren’t competitors, but equally legitimate models of *different* positively conceived mental health phenomena. Accepting pluralism steers us towards such avenues of enquiry in the scientific domain.

Finally, what about the *practical* implications of pluralism? Let’s consider just one issue: the implications for psychotherapy. Consider the question of what makes a psychotherapeutic intervention *therapeutic*. I don’t mean by this the question of what therapeutic school or modality it belongs to. Or the question of how it works or is effective. The question I am asking is what do we *mean* in describing an intervention as therapeutic.

A natural answer here is that an intervention is therapeutic insofar as it aims at supporting mental health. Now, if we say this, and accept mental health pluralism, this opens up an interesting possibility: the possibility of *psychotherapeutic pluralism*. We can understand this as the view that different psychotherapeutic interventions can be therapeutic in different ways. Not in the familiar sense that they belong to different modalities or schools, or employ different means and mechanisms of therapeutic change. But in the sense that there isn’t a single concept of *the therapeutic*, aligned with a single concept of mental health, but rather multiple concepts of the therapeutic aligned with multiple concepts of mental health.^33^

Why does acknowledging this possibility matter? After all, we already acknowledge plurality in the world of psychotherapy—e.g., different modalities.^34^ And this is helpful to people who seek mental health support, as it can help people to fit things to their specific needs and personality.

However, acknowledging such plurality is consistent with viewing different therapeutic approaches as directed at a single phenomenon: mental health. From a psychotherapeutic pluralist perspective, thinking of things in this way is bound to foster an overly narrow understanding of the power of psychotherapy. For example, if one accepts the popular idea that mental health is simply the absence of mental disorder, one might not appreciate the ways in which various modalities of psychotherapy can help support one in overcoming *non-pathological* psychological problems, and the ways in which psychotherapy can help support one in developing one’s mental health *positively* conceived: promoting growth, self-understanding, and a range of positive emotions.

Considering the prospects for psychotherapeutic pluralism matters, then, since if it is correct, there is a further layer of plurality in psychotherapy that we can highlight to help people even more in finding support that fits them: support that accords with therapy in a sense that is appropriate to them, and mental health in a sense that is appropriate to them.

## Footnotes

1 Indeed, the question of how to define mental health has received relatively little attention in philosophy especially when compared with related concepts such as mental illness or disorder. Notable exceptions include Macklin (1972), Boorse (1976), Brown (1977), Engelhardt and Spicker (1978), Tengland (2001), Keller (2020), and Wren-Lewis and Alexandrova (2021).

2 See also Manwell et al. (2015).

3 Some of these comparisons reflect the structurally different health concepts we operate with. These include but aren’t limited to health as freedom from illness, health as the ability to function, health as fitness, and health as a reserve (Blaxter 1990, p. 16). See also Sholl and Rattan (2020) for a discussion of various concepts of health.

4 This is not to say that there are no existing anti-pluralist views. The point is that because of the neglect of the question of pluralism in the literature, it is often unclear whether or not existing views on the nature of mental health should be understood in pluralist or anti-pluralist terms. Some do proceed *as if* there is a single phenomenon or concept of mental health in play (e.g., Tengland 2001, and Wren-Lewis and Alexandrova 2021). But this doesn’t mean that they reject pluralism. The question of pluralism is not on the table in such discussions, and so it is difficult to say whether they take their analyses to be of *the* phenomenon of mental health, or just one of potentially many mental health phenomena.

5 Such plurality comes across well in collections on mental health such as Reynolds et al. (2009) and Bhugra et al. (2018), and in textbook treatments such as Pilgrim (2023). See also Mirowsky and Ross (2017) on mental health and development, and Richter and Dixon (2023) on mental health models and measures.

6 For similar reasons, ‘medical pluralism’ (Khalikova 2021) is not sufficient for mental health pluralism.

7 On the difference between ‘constitutive’ and ‘threshold’ dependence, see Alexandrova (2017, p. 8).

8 Though I am yet to find explicit proponents of this specific view in philosophical literature, I interpret Jahoda (1958, pp. 66–73) as accepting a similar view, as she emphasises the diversity of both concepts and types of mental health. See also Hartmann (1981, p. 365) who reminds us that ‘theoretical standards of health are usually too narrow in so far as they underestimate the great diversity of types which in practice pass as healthy.’ For pluralist conceptions of health more generally see Nordby (2006, 2019), Haverkamp et al. (2018), Valles (2018), van der Linden and Schermer (2021), Binney et al. (2024), Kukla (2024), and De Vreese (2024).

9 C.f., Nordenfelt (1995, p. 6) and Mitchell and Alexandrova (2021, p. 2416).

10 See also Nordenfelt (1995, pp. 6–7) who offers a substantive philosophical analysis of health, yet aims merely to capture our conventional understanding, which he distinguishes from essentialist approaches. Tengland (2001) applies this to mental health.

11 I understand concepts such that if ‘mental health’ varies in its sense/meaning/semantic value/content across contexts, then it expresses different *concepts* of mental health across those contexts.

12 See: WHO (2022).

13 See: WHO (n.d.).

14 Similarly, see Tengland (2001, p. 16).

15 Foregrounding the question of *well-being* pluralism will no doubt introduce further ambiguities too. Since my main concern is with mental health, I leave this aside. Ultimately, one might endorse pluralism about mental health and about well-being.

16 Jahoda (1958, p. 8) draws a similar distinction between mental health as ‘an enduring attribute of a person’ and mental health as a ‘momentary [i.e. relatively temporary] attribute of functioning’.

17 See, e.g., Stuifbergen et al. (1990) and Lindsey (1996).

18 Positive psychology conceptions of mental health explicitly make room for the co-instantiation of mental health and (so-called) mental illness, see Delle Fave and Negri (2021).

19 For another example, consider the debate between *naturalists* (e.g., Boorse 1976, 1977) and *normativists* (e.g., Reznek 1987, Nordenfelt 1995, and Cooper 2002). Crudely, naturalists hold that health and disease can be analysed in objective, value-free, biological terms (e.g., in terms of biological function/dysfunction). Whereas normativists reject this, and hold that health and disease are thoroughly value-laden (for helpful critical overviews of this dispute, see Nordenfelt 2006 and Kingma 2019). To apply what I’ve argued, we can observe that any naturalist or normativist account of mental health is inherently unclear or ambiguous unless it is related to the question of pluralism. For unless it is so related, we won’t know whether it is to be understood in anti-pluralist terms or pluralist friendly terms—and if pluralist friendly, whether it applies to all or just some forms of mental health (assuming there are multiple forms). Indeed, embracing pluralism opens up the potential for embracing *both* views (and not in the hybrid way that Wakefield (1992) embraces both views): if, that is, it turns out that some forms of mental health are best understood in naturalist terms, yet *other* forms of mental health are best understood in normativist terms. Unfortunately, the question of pluralism is largely neglected in the debate between normativists and naturalists. One exception is Boorse. Though he doesn’t explicitly formulate the question of pluralism, he does seem to embrace a form of it, as he is clear that his value-free naturalist analysis is intended to apply only to a *certain* conception of health: *theoretical health*. He understands this in terms of the absence of disease, where this, in turn, is understood as a biological dysfunction. But in addition, he recognises *practical health*: a ‘value-laden practical counterpart’ (Boorse 1997, p, 97), which he initially understood in terms of the absence of illness (Boorse 1975), but later in terms of other concepts which reflect the values of medical practice, namely diagnostic normality (the absence of clinically detectable pathology), and therapeutic normality (the absence of clinically treatable pathology) (Boorse 1987, 2014).

20 I am indebted to Alexandrova (2017) who makes a similar argument with respect to well-being pluralism.

21 Elements of this example are drawn from Inada et al. (2020).

22 In Alexandrova (2017, p. 8)’s terminology, there seems to be a difference in what mental health constitutively depends on.

23 I’m assuming that in our specific contexts the psychiatrist isn’t concerned with whether Fabio has the kinds of states that the trainer focuses on (and so I’m assuming that their absence isn’t indicative of mental disorder), and that the trainer isn’t concerned with the disorder status that the psychiatrist focuses on. In other contexts, of course, such factors might be closely connected.

24 C.f., Circumscription views of well-being (Alexandrova 2017, p. 5).

25 C.f., Alexandrova (Alexandrova2017, pp. 8–10)’s worries about Circumscriptionist views.

26 This is akin to versions of monism about well-being that accept what Alexandrova (2017) calls ‘Differential Realisation’, or what Mitchell and Alexandrova (2021) call ‘constitutive pluralism’.

27 The contexts differ in what is relevant to mental health partly because of the differences in practical concerns/goals, and relationships: e.g., treatment vs moral support, doctor vs friend. (C.f., the differences in the context of palliative care and urban planning discussed earlier).

28 One might interpret Nordenfelt (1995, pp. 105–112) as endorsing this kind of view, since he claims that the reference class, but not the content, of a health ascription is sensitive to social context. Alexandrova (2017, p. 11) helpfully notes how in the case of well-being such a view is similar to subject-sensitive invariantist positions that we find in epistemology.

29 And here one might worry that what has been offered is an *intuitive* rather than a *technical* case. We haven’t considered whether contextualism about ‘mental health’ is technically possible or plausible by looking at the linguistic side of things. However, I am optimistic on the technical front, as mental health vocabulary seems to satisfy sensible linguistic criteria for context-sensitivity in just the way that well-being vocabulary does (see Alexandrova 2017, pp. 14–16, drawing on Stanley 2004, and Cappelen and Lepore 2005).

30 This point also applies to the account developed by Tengland (2001), given its similarity to that of Wren-Lewis and Alexandrova (2021). It also applies to a ‘disjunctive’ understanding of the essence of mental health on which it is *either being free of mental disorder, or having certain positive psychological states.*

31 I think the ideas of this essay can also be adapted to support a more general *health* pluralism, but I leave discussion of this for another occasion.

32 C.f. Alexandrova (2017, p. 22).

33 This assumes that there would be more to being therapeutic than can be extracted from whatever structural core is common to multiple mental health phenomena.

34 Indeed, there is even an emphasis on plurality *within* certain psychotherapeutic approaches, most obviously in the approach known as ‘pluralistic psychotherapy’. This approach invovles ‘three pillars of pluralism’ such that a pluralistic practitioner (1) is open to a variety of psychotherapeutic *orientations* and methods, rather than those of just one particular school, (2) acknowledges and celebrates diversity across *clients*, and (3) emphasises the different *perspectives* of therapist and client in the therapeutic process (Cooper and Dryden 2016, p. 3). However, this approach doesn’t entail the further level of plurality that I am highlighting in outlining psychotherapeutic pluralism: wherein there are many forms of mental health which ground many ways of being therapeutic. For one could still endorse pluralistic psychotherapy, and emphasise the plurality of orientations, clients, and perspectives even if one holds that all psychotherapy (even pluralistic psychotherapy) ultimately aims at a single substantive thing, mental health.
